# Calcium Imaging of GPCR Activation Using Arrays of Reverse Transfected HEK293 Cells in a Microfluidic System

**DOI:** 10.3390/s18020602

**Published:** 2018-02-16

**Authors:** Margriet Roelse, Maurice G.L. Henquet, Harrie A. Verhoeven, Norbert C.A. de Ruijter, Ron Wehrens, Marco S. van Lenthe, Renger F. Witkamp, Robert D. Hall, Maarten A. Jongsma

**Affiliations:** 1 BU Bioscience, Wageningen University and Research, Droevendaalsesteeg 1, 6708 PB Wageningen, The Netherlands; maurice.henquet@wur.nl (M.G.L.H.); harrie.verhoeven@wur.nl (H.A.V.); ron.wehrens@wur.nl (R.W.); robert.hall@wur.nl (R.D.H.); maarten.jongsma@wur.nl (M.A.J.); 2 Laboratory of Plant Physiology, Wageningen University and Research, 6708 PB Wageningen, The Netherlands; 3 Laboratory of Cell Biology, Wageningen University and Research, 6708 PB Wageningen, The Netherlands; norbert.deruijter@wur.nl; 4 BU Biometris, Wageningen University and Research, 6708 PB Wageningen, The Netherlands; marco.vanlenthe@wur.nl; 5 Human Nutrition and Health, Wageningen University and Research, Stippeneng 4, 6708 WE Wageningen, The Netherlands; renger.witkamp@wur.nl

**Keywords:** reverse transfection, cell array, GPCR, NK1 receptor, Cameleon YC3.6, microfluidics

## Abstract

Reverse-transfected cell arrays in microfluidic systems have great potential to perform large-scale parallel screening of G protein-coupled receptor (GPCR) activation. Here, we report the preparation of a novel platform using reverse transfection of HEK293 cells, imaging by stereo-fluorescence microscopy in a flowcell format, real-time monitoring of cytosolic calcium ion fluctuations using the fluorescent protein Cameleon and analysis of GPCR responses to sequential sample exposures. To determine the relationship between DNA concentration and gene expression, we analyzed cell arrays made with variable concentrations of plasmid DNA encoding fluorescent proteins and the Neurokinin 1 (NK1) receptor. We observed pronounced effects on gene expression of both the specific and total DNA concentration. Reverse transfected spots with NK1 plasmid DNA at 1% of total DNA still resulted in detectable NK1 activation when exposed to its ligand. By varying the GPCR DNA concentration in reverse transfection, the sensitivity and robustness of the receptor response for sequential sample exposures was optimized. An injection series is shown for an array containing the NK1 receptor, bitter receptor TAS2R8 and controls. Both receptors were exposed 14 times to alternating samples of two ligands. Specific responses remained reproducible. This platform introduces new opportunities for high throughput screening of GPCR libraries.

## 1. Introduction

Reverse transfection is a technique to create arrays of transiently-transfected cells on a solid surface. Since the first publication of Ziauddin and Sabatini in Nature in 2001 [1], the technique has found versatile application addressing different cellular and biological questions using printed cDNAs, siRNAs, viruses and even chemical compounds [2,3,4,5,6,7].

**Figure 1:**
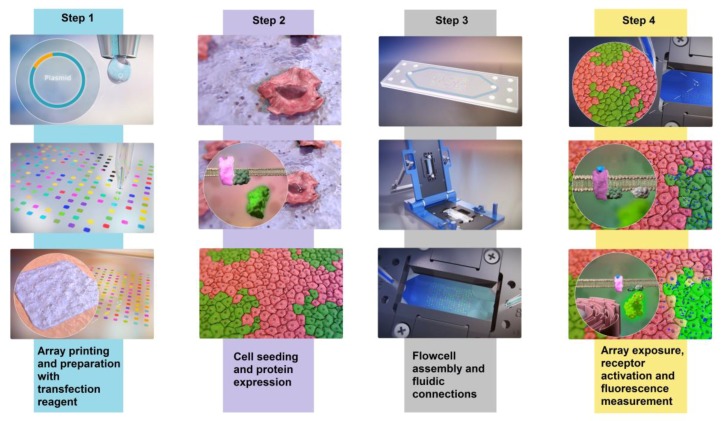
Schematic overview of array preparation and measurement in the microfluidic system. Steps 1 and 2 show array preparation involving DNA spot contact printing, overlay with transfection reagent, subsequent seeding of cells and expression of DNA after cell division. Step 3 shows the assembly of the flowcell and placement into the Micronit FluidConnect Pro flowcell holder. Once the holder is closed, fluidic connections are established, and the microfluidic system produces a pressure-controlled flow. Specific volumes of samples are injected sequentially into the constant flow to stimulate cell responses on the micro array (Step 4). Continuously, the entire array within the flowcell is imaged to capture the fluorescence from the calcium indicator YC3.6 over time and allow high-throughput screening. See also Supplementary Materials Video S1 and Figure S1.

The generation of cell arrays through reverse transfection using plasmid DNA involves three basic steps: (i) Printing a DNA micro-array; (ii) coating the DNA microarray with transfection reagents; and (iii) seeding with adherent cells to cover the pre-treated DNA micro-array. Steps (i) and (ii) are sometimes combined [4,8,9], but typically, these three basic steps can transform a printed DNA microarray into a transfected cell array via efficient contact-mediated uptake of plasmid DNA by the adhering cells. This is visualized in Figure 1, Steps 1 and 2, Video S1 and Figure S1. Protein expression efficiency depends, to a great extent, on correct formation and delivery of DNA transfection complexes. These aspects have been studied for both surface-immobilized and solution-mediated transfection systems [10,11,12]. Other important parameters influencing protein expression efficiency are the cell cycle [13,14], the concentration [15,16,17] and the purity of plasmid DNA [18]. Higher plasmid concentrations have a direct effect on intracellular plasmid copy numbers in both the cytoplasm and the nucleus up to a certain maximum. Cohen et al. [15] have shown that with increasing DNA levels, the maximum gene expression depends on cell type, the vector used and the method of transfection, so that no single protocol can be applied in all situations.

Only a few studies have combined reverse transfection with microfluidics [19,20,21,22]. In those studies, each spot of the cell array was enclosed by a chamber that allowed individual fluidic control. These micro-chambers mimic a multi-well plate where each well or chamber is individually used for measurements. However, a setup applying controlled fluidics to individual spots is relatively complex for generic screening of GCPR libraries. This merited developing a system allowing simultaneous exposure of multiple receptors within a single chamber, thereby simplifying the setup and increasing the efficiency of screening and analysis. In this article, we show how we have developed such a screening system involving an array in a single flowcell and a stereomicroscope to simultaneously image all spots over time (Figure 1, Steps 3 and 4, Video S1 and Figure S1).

Reverse-transfected cell arrays have been used before to screen GPCR libraries, but those arrays were small and spotted in the static environment of 96-well plates [23,24]. These setups did not allow for a sequential microfluidic screening of samples. The latter enables not only a dynamic analysis of receptor-ligand interaction, but also a more powerful spot-based, statistical analysis of sequential injections. In the present study, we placed a reverse transfected cell array in a re-sealable microfluidic chamber that covered the entire array (Figure S1). This setup allowed routine monitoring of the ionic calcium responses of each individual spot to sequentially-injected samples. As a proof-of-concept, we used the NK1 receptor, which was previously characterized in this flowcell [25,26], and the bitter taste receptor hTAS2R8 [27]. The activation of these receptors by their respective ligands led to a transient increase in the cytoplasmic calcium ion concentration via a signal transduction pathway involving uncoupling of the G proteins, activation of phospholipase C, inositol trisphosphate (IP3) production and opening of an IP3-gated calcium channel of the endoplasmic reticulum [28,29]. Such calcium responses are transient and were monitored in real time using the fluorescent calcium-ion sensor protein Cameleon YC3.6 [30,31,32] by means of FRET imaging. Previously, we expressed the NK1 and YC3.6 genes from a single plasmid. Here, we have designed and applied a more flexible system using separate plasmids for each gene. This enabled the modulation of receptor gene expression, independently of calcium sensor expression, by modifying plasmid DNA ratios. In this paper, we focused on the parameters of the reverse transfection procedure to optimize GPCR response in our microfluidic system and on the appropriate data analysis. In such a system, the following properties are paramount for optimal use: protein expression efficiency; transfection uniformity over the array; co-transfection and a reproducible response to repeated challenges (robustness). The potential of this system for high throughput screening of GPCR receptor libraries is discussed.

## 2. Materials and Methods 

An animation of the array preparation, flowcell assembly and array measurement is presented in Supplementary Materials Video S1 and Figure S1. 

### 2.1. Expression Vectors 

The NK1 receptor gene was purchased at the UMR cDNA Resource Center (Bloomsburg University, catalog number #TACR100000, Bloomsburg, PA, USA). The hTAS2R8 (NCBI Reference Sequence: NM_023918.1) bitter receptor gene was PCR amplified from genomic DNA from HEK293 cells and cloned into a pcDNA3 expression vector. This vector contained a DNA sequence encoding the first 45 amino acids of the rat somatostatin receptor type 3 to improve membrane targeting of the bitter receptor protein. The Ga16GUST44 gene in vector pcDNA3 was a gift from Dr. Takashi Ueda (Nagoya City University, Nagoya, Japan). The YC3.6 gene in pCDNA3 (ThermoFisher scientific, Waltham, MA, USA) was a gift from Prof. Roger Tsien (UC San Diego). Plasmids containing CFP and RFP were from Addgene (#13030 and #13032, respectively). All plasmids used were of similar size and carried the same CMV promotor.

### 2.2. Cleaning and Printing of DNA Arrays 

Glass slides (15 × 45 mm) were cleaned with 2% Hellmanex solution (Hellma Analytics, Müllheim, Germany) by dipping the slides in the solution, sonication for 15 min and subsequent incubation for a maximum of 2 h at room temperature. The slides were then thoroughly rinsed and incubated overnight at room temperature in ultra-pure water to remove any residue left by the Hellmanex solution. After incubation, the slides were dried in a 50 °C incubator and coated for one hour with poly-l-lysine 100 µg/mL solution (Sigma, St. Louis, MO, USA, P1274). The slides were then rinsed with ultra-pure water, dried and stored at room temperature in a dust-free container until use. The slides were printed by a Microgrid II DNA printer (BioRobotics) using modified SMP3 pins (arrayit), which were slightly blunted to generate square ~200 µm × 200 µm spots (Figure S2B). Slides were printed at ~55% relative humidity and left to dry for one hour after printing. The spots were initially printed using a 400-µm grid spacing, but as the resulting transfected cell spots appeared at risk of cross-contamination because of slight cellular motility, spot spacing was subsequently increased to 600 µm (Figure S2D). The print solution was composed of purified plasmid DNA (Qiagen, Plasmid Mini kit, 12123, Germantown, USA), 0.4% gelatin (Sigma, G9391, Zwijndrecht, Netherlands) and 50 mM sucrose dissolved in ultra-pure water. The gelatin was prepared fresh before use by dissolving 1% gelatin in ultra-pure water at 60 °C for 15 min and filtering it through a 0.2-µm filter. After printing, the slides were stored at room temperature in a dust-free and dry container. Slides could be stored for up to 6 months without loss of transfection yields. Print solutions were freshly made, but can be stored at −20 °C and re-used after adjusting volumes and heating to 60 °C for 15 min.

### 2.3. Cell Culture and Reverse Transfection 

HEK293H cells (ThermoFisher Scientific, Waltham, MA, USA, 11631-017) were maintained as a monolayer in DMEM (Gibco, 21063-029) containing 1× MEM non-essential amino acids (Gibco, 11140-035), 1× penicillin-streptomycin (Gibco, 15140-122) and 10% FBS (Gibco, 26140) in a 37 °C incubator with 5% CO_2_. HEK293H cells stably expressing the chimeric G protein subunit Ga16gust44 [33] were used for the expression of hTAS2R8. The reverse transfection method was adapted from the Sabatini lab description [1]. In short, we prepared a transfection mix from the Effectene kit with transfection reagents (Qiagen, 301425) by mixing 75 µL EC buffer with 8 µL enhancer, pre-incubating for 5 min at room temperature and then adding 12.5 µL Effectene lipids. The transfection mix was vortexed for 10 s and carefully applied, dropwise, onto the glass surface submerging the entire 15 × 18 spot array. The slides were incubated for 20 min at room temperature. The transfection mix was removed by tilting the array and carefully pipetting off the transfection mix. Once the slides were air-dried, a flexiPERM chamber (Greiner, 90032039) that was cut to the appropriate size was mounted over the array (Figure S1A). The 0.9-cm^2^ chamber was filled from the edge with HEK293H cells (2.2 × 10^5^ cells/cm^2^) and incubated at 37 °C with 5% CO_2_ for at least 27 h before use.

### 2.4. Flowcell and Array Imaging Setup 

**Figure 2:**
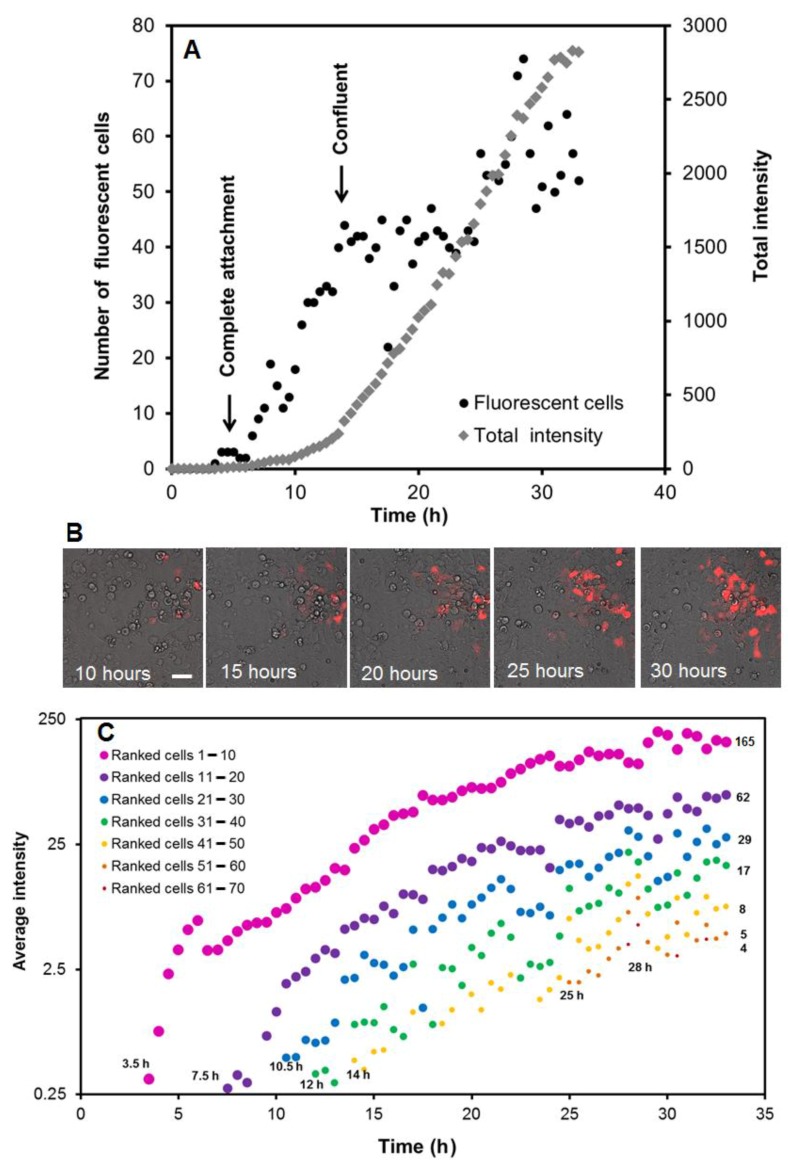
Time course of RFP expression after reverse transfection. (**A**) Graph of the number of fluorescent cells (left axis, black dots) and the total fluorescence of all cells (right axis, open diamonds) in time. Based on bright field images, the complete cell attachment and the moment when complete confluence of the cell layer was reached are indicated in the figure. (**B**) Overlay of bright field and RFP fluorescence at 10, 15, 20, 25 and 30 h after inoculation. Scale bar = 50 µm. (**C**) Graph of the average intensity within pools of ranked fluorescent cells over the time course of the reverse transfection. The pools represent up to 10 fluorescent cells ranked from the highest to lowest expressing cells at each timeframe. The start time of each pool is indicated in the graph, and the average intensity of the pool at 33 h post transfection is shown on the right within the graph.

The reverse transfected arrays (270 spots and ~100 mm^2^) were mounted with a resealable borosilicate glass flowcell (Micronit Microfluidics B.V, Resealable FC 4515, Extended 10 mm) and fitted in a flowcell holder (Micronit Microfluidics B.V., Fluidic Connect PRO Chip Holder with 4515 Inserts, Figure S1A). The arrays were imaged by a Leica fluorescent stereo microscope (Leica M205FA with DFC 345 FX camera and 2.0× PlanApo objective with NA 0.35) fitted with a 0.32× C-Mount and filters for CFP (ET CFP 10447409, excitation 436/20 and emission 480/40) and YFP FRET (ET FRET 10450566, exc. 436/20 and em. 535/30). Lamp intensity (Osram, HXP-R 120W/45C VIS) was set at maximum, and the exposure time was 400 ms. Large cell arrays as shown in Figures S1B and S3 were imaged at the maximal field of view (FOV) in order to image the fluorescent signal from the entire array. Compared to the confocal microscope images of Figure 2B (~0.16 mm^2^), this led to a loss of cellular resolution due to the larger working distance, but a ~625× larger area that was imaged. From Figure S3B, it is evident that due to this zooming out, cellular contours were lost and that a single pixel of about 11 μm in diameter can cover one or partially two cells. In the time series acquisition, a 2× binning was used resulting in a 23 × 23-μm pixel size on chip, which corresponds with single HEK cells in the sample. The variation in fluorescent protein expression between cells is therefore correlated with the variation in individual pixel intensity. The graph in Figure S3C shows the pixel intensity against the ranked pixels of the enlarged section. At 8-bit resolution, pixels with a fluorescence intensity of 256 have reached the detector limit. Pixels that represent the background in the selection (e.g., at the edge of this section) have a background intensity of 0~20, and pixels with intensities between ~20 and 256 represent those with fluorescent protein expression. To distinguish fluorescent pixels from the background, we set a lower limit (threshold) using the background algorithm in the CellProfiler software. On large arrays expressing the receptor proteins, calcium responses from receptor activation were imaged in time. A continuous flow of 100 µL/min culture medium or microscopy buffer (NaCl 130 mM, KCl 5 mM, glucose 10 mM, CaCl_2_ 2 mM, HEPES 10 mM at pH 7.4) was applied for 10 min before the samples were injected. Sample injections were done using a manual injection valve (Upchurch, V-451) containing a 50-µL loop. Channel gains were maintained after initial separate adjustment for CFP and YFP FRET channels to measure fluorescence intensity amplitudes and minimize pixel saturation of the reverse transfected array. Images were taken every 3–5 s (Figure S1B). 

### 2.5. Live Imaging of Reverse Transfection Time Course 

A series of images of the reverse transfected cell array was taken to monitor cell motility and fluorescence intensity in time. Minimal light stress during acquisitions was achieved by spinning disk confocal imaging (Andor-Revolution system with Yokogawa disk) on a Nikon Ti Eclipse inverted microscope equipped with an autofocus (PFS3), piezo stage (ASI XY-LE) and EMCCD camera (Andor Ixon888, 1024 × 1024 pixels, 13 × 13 µm). Optimal physiological conditions were maintained by a stage top incubator (Tokai Hit, Shizuoka, Japan) at 37 °C ± 0.1 °C with continuous humidified 5% CO_2_ flow. Images were acquired every 30 min over a period of 33 h with a 10× CFI Plan-Fluor NA 0.3. Signal expression from CFP, YFP or RFP was monitored in corresponding domains of the reverse transfected cell array. Images were recorded with MetaMorph Imaging Series 7.8.3 (Molecular Devices, San Jose, CA, USA).

### 2.6. Data Analysis of Fluorescent Protein Expression

Pixel intensity data were obtained from the Leica software (LAS AF Lite, Version 3.1.0, Leica Microsystems B.V., Amsterdam, Netherlands). A grid of equally-sized regions of interest (ROI) was created, each containing one array spot. The intensity histogram of each ROI was then exported to a spreadsheet program. There, the pixels were sorted against the intensity value and numbered, starting with the highest intensity value. When ranked pixels from different reverse transfection conditions (e.g., input DNA concentration) were compared, a ranking range was chosen that yielded a value between the upper and lower thresholds for each condition. To avoid skewed data comparisons, only pixels in this dynamic range rather than an ROI average were used to yield a valid analysis of DNA concentration effects.

### 2.7. Data Analysis of Calcium Responses 

The images recorded on the Leica fluorescence stereo microscope were first analyzed using Cell Profiler software Version 2.1.1 (freeware) [34]. Two CellProfiler pipelines were created. In the first pipeline, one YFP image was used to create a mask of the array. This mask separates signal pixels from background pixels and is based on the threshold algorithm given in the pipeline. In the second pipeline, a grid overlay was created to define the array spot positions. The mask image from the first pipeline was used as the overlay on every CFP and YFP image of the recorded time series. The intensity values of the pixels within the mask for both CFP and YFP images were measured, and the signal was defined by calculating YFP/CFP ratios. Peak heights were then determined for all spots and all injections by measuring the range of the signal within the first 40 cycles. In order to be able to compare receptor responses to different types of injection, a linear mixed model was constructed relating the injection type to the receptor type. Since different spots of the same receptor type can sometimes have quite different baseline responses, the spot number was taken into account as a random factor. The outcome of the model consists of estimated responses (including confidence intervals) of each receptor type to all injection types. The estimated differences with a reference injection (here the blank) can then be visualized, including confidence intervals calculated for each injection type separately. Model calculations were performed using in-house software (publication in preparation).

## 3. Results

### 3.1. Time Course of Protein Expression after Reverse Transfection at the Cell and Spot Level 

Protein expression of fluorescent proteins was followed live and used to establish the time-course of protein accumulation both at the single cell and the reverse-transfected spot levels. Fluorescence emission of a reverse transfected spot carrying the RFP plasmid DNA was imaged using a spinning disk confocal fluorescence microscope at 30-min intervals for a period of 33 h (Figure 2, Video S2). Imaging was initiated directly after seeding the cells on top of a DNA array pre-treated with Effectene. Cells became fully attached to the surface in ca. 5 h, initially covering about 50% of the slide. At 3–5 h post cell inoculation, the first cells started to show detectable fluorescence above background levels. To follow this in detail, we counted the number of transfected cells at each time point and measured the total fluorescence intensity of the image (Figure 2A). It should be noted that accurate counting of fluorescent cells was difficult, because these tended to overlap and move between images, which is a sign of vitality in HEK293 cells [35]. Together with the effects of cell division, this did result in fluctuations of the number and intensity of (ranked) cells (Figure 2A,C). The total fluorescence of the image showed a nearly linear increase between 14 and 30 h, post inoculation (Figure 2A). We also observed a large variation in fluorescence intensity between the transfected cells as shown in Figure 2B, even though the spot was transfected with a fixed RFP-encoding plasmid DNA concentration. The range in fluorescence intensity between individual cells increased over time. To understand this, we ranked the fluorescence intensities from high to low for each time point and plotted the average intensities in pools of 10 cells per rank, also from high to low, as shown in Figure 2C. We observed that the fluorescence of cells did not decrease in time nor increase faster when detected later. Thus, the figure shows that cell groups with early expression of RFP indeed reach the highest final level. The delay in visible RFP expression in subsequent classes of cells is not only characterized by a time delay, but also by lowered maxima. For example, a 4-h delay in expression of the first two classes resulted in a circa 2.5-fold lower RFP expression level after 30 h and a 10-h delay in a ~30-fold lower expression. Since the variation in RFP expression within the reverse-transfected spot was large, we investigated, given the intercellular variation in the expression of proteins, how large reverse-transfected DNA arrays could best be imaged by fluorescence stereomicroscopy and how expression relates to the DNA dosage.

### 3.2. Effect of DNA Concentration on Protein Expression

**Figure 3:**
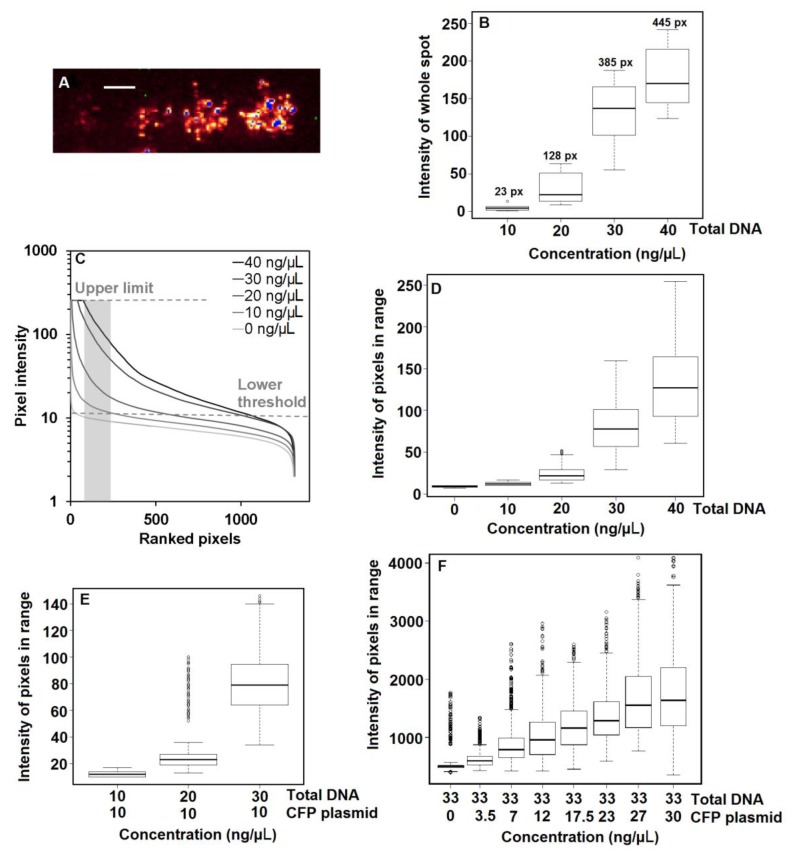
The effect of total DNA concentration and coding DNA concentration on protein expression displayed as pixel intensity. The boxplots of Figure 3 show the distribution of spot intensities per category. Each category includes about 10 spots. The black line indicates the median value of the data distribution; the box represents the first and third quarter of the data distribution; and the whiskers represent the minimum and maximum value of the dataset. The open circles are outliers in the dataset. (**A**) Fluorescence image of four spot types representing from left to right 10, 20, 30 and 40 ng/µL total DNA. The image is shown with a range indicating LUT where over-exposed pixels display in blue. Scale bar = 200 µm. (**B**) Average spot intensity (based on pixels above background intensity including saturated pixels) of spots containing total DNA at concentration of 10 ng/µL (*n* = 9 spots), 20 ng/µL (*n* = 8 spots), 30 ng/µL (*n* = 9 spots) and 40 ng/µL (*n* = 8 spots). Numbers above the boxplots indicate the average amount of pixels of the spots. (**C**) Pixel ranking of spots containing 0 ng/µL, 10 ng/µL, 20 ng/µL, 30 ng/µL and 40 ng/µL total DNA. The grey dashed lines show an upper limit (with a value of 256) and a lower threshold (background level with a value of ~20). The grey area (Pixels 110–212) is the range of pixels where all spots can be compared using the same pixel range and within the upper and lower limits. (**D**) Boxplots of CFP fluorescence intensity of all pixels in range (grey bar) as indicated in (C) with increasing total CFP plasmid DNA concentration. (**E**) Boxplots of CFP fluorescence intensity of all pixels in range with 10 ng/µL (*n* = 9 spots), 20 ng/µL (*n* = 9 spots) and 30 ng/µL (*n* = 9 spots) total DNA and constant 10 ng/µL CFP plasmid DNA. (**F**) Boxplots of CFP fluorescence intensity of all pixels in range with increasing CFP plasmid DNA concentration and constant total DNA concentration. The array of Figure 3F was measured on a different array (all conditions *n* = 26 spots) with a 12-bit intensity scale (4096 levels), leading to higher intensity values and a different pixel range for intensity comparison than (**D**,**E**).

To determine the effect of DNA concentration on protein expression, an array was prepared using various concentrations of CFP plasmid DNA. A fluorescence image of a representative part of this array is shown in Figure 3A, with over-exposed pixels in blue. Like Figure 1, each spot in Figure 3 contains pixels with a wide range of fluorescence intensity levels, which reflects the differences in cellular expression levels. In these recordings, the detector gain was deliberately set to allow some saturation at the highest expressing cells in order to keep the low expressing cells within the detection range. With increasing DNA concentration, an increase in both fluorescence intensity and number of pixels per spot was observed (Figure 3B and Figure S2), which also increased the number of saturated pixels. If saturated pixels were included in an analysis of the effect of different total DNA concentrations, expression efficiency would be underestimated. The other way around, leaving out underexposed pixels with no value leads to overestimation of expression efficiency. This is shown graphically in Figure 3C using a pixel ranking of all pixels in spots transfected using 0–40 ng/µL CFP plasmid DNA. In this plot, the lower threshold separates the signal from background so that spots generated with 0 ng/μL DNA show no fluorescence. Fluorescence of CFP spots was measured up to an intensity value of 256, the limit of the detector. The grey bar in Figure 3C shows a range of comparable, ranked pixels with non-saturated fluorescence above the lower threshold at all four concentrations tested. For these pixels, intensity reports the direct relationship between protein expression and DNA concentration.

In Figure 3D, boxplots of all pixel intensities within the pixel range of Figure 3C are shown. A two-fold increase in CFP fluorescence is observed from 10 and 20 ng/µL, but between 10 and 40 ng/µL, there is almost a ten-fold increase in CFP expression, suggesting that the relationship between protein expression and total DNA concentration is not linear. Since standard deviations increase with increasing DNA concentration, a linear relationship with the log-transformed CFP intensities can be expected. This indeed leads to a high R2 value of 0.89 (compared to 0.68 for the untransformed CFP intensities).

### 3.3. Effect of Co-Transfection on Protein Expression

To investigate non-linear effects of DNA concentration on protein expression, two arrays were prepared keeping either the CFP plasmid DNA concentration or the total DNA concentration constant. With a constant CFP plasmid DNA concentration, the dependency of cell transfection on total DNA would be established, and with a constant total DNA concentration, the dependency of CFP expression on DNA plasmid copy number would become visible. Figure 3E shows the intensity of CFP fluorescence with increasing total DNA concentration, but at a constant CFP plasmid DNA concentration of 10 ng/µL. This figure follows the same pixel intensity increase as in Figure 3D. This indicates that, in this concentration range, CFP expression almost entirely depended on the total DNA concentration rather than the specific CFP plasmid DNA concentration. Figure 3F shows the intensity of CFP fluorescence with 33 ng/µL total DNA concentration and an increasing proportion of CFP plasmid DNA. Despite the large variation, there was a linear relationship between the concentration and the average CFP intensity, described by the following formula:

pixel intensity = 39.6[CFP] + 534

This indicates a background fluorescence of 534 and a slope coefficient of 39.6 for the increase of CFP fluorescence in this array, with an R^2^ value of 0.985. Also at the highest DNA concentration, no clear saturation effects were visible.

### 3.4. Effect of DNA Concentration on NK1 Receptor Calcium Signaling 

Considering the observed effects of DNA concentration on protein expression, we next studied the effects of the coding DNA concentration on the functional activation of the G-protein coupled receptor neurokinin 1 (NK1). The transient cytoplasmic calcium signal was measured with the co-transfected calcium sensor protein Cameleon YC3.6. All spots were transfected with the same amount of calcium sensor resulting in similar total fluorescence levels between spots. The optimum gain was set to maximize the number of fluorescent pixels. This led to some overexposed pixels, which were omitted from the analysis.

**Figure 4:**
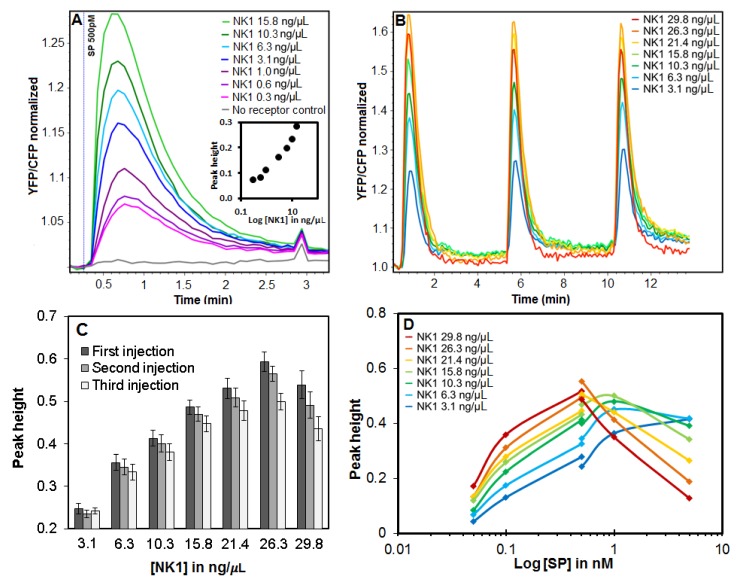
Calcium ion dynamics after substance P (SP) exposure to arrays co-transfected with the NK1 receptor and YC3.6 calcium sensor. Calcium ion dynamics are displayed in the YFP/CFP signal ratio obtained from the YC3.6 calcium sensor and normalized by dividing the CFP and YFP signals by the average of the first images before sample exposure. (**A**) Cytosolic calcium ion transients after 500 pM SP exposure rise with increasing NK1 plasmid DNA concentration ranging from 0.3 ng/µL–15.8 ng/µL, while total DNA concentration was kept at 33.3 ng/µL. The inset chart shows a correlation between NK1 plasmid DNA concentration and peak height. (**B**) Repeated injections of 500 pM SP on an array with a total DNA concentration of 33.3 ng/µL and NK1 plasmid DNA concentration ranging from 3.1 ng/µL–29.8 ng/µL. (**C**) Bar chart of (B) showing the peak height and standard error for each injection of 500 pM SP at the different NK1 plasmid DNA concentration. (**D**) Dose response curve for the NK1 receptor and SP ranging from 50 pM–5 nM SP. The curve was measured over two arrays as shown by the interruption in the curves. All values in this figure represent an average of over 20 spots each, distributed equally over an array.

The first question we addressed was the relationship between receptor DNA concentration (ranging from 0.3 ng/µL–15.8 ng/µL in print solutions of 33 ng/µL total DNA) and the receptor activation response. Transfected arrays were assembled in a microfluidic flowcell with a continuous flow of 100 µL/min culture medium. Samples of substance P (SP), an 11-amino acid neuropeptide that activates the NK1 receptor, were injected using a 50-µL sample loop, resulting in a transient exposure to SP of about 30 s. Fluorescence emission of the calcium sensor protein was ratio-imaged every 5 s. After 2 min, the cytosolic calcium ion level had returned to basal levels, indicating a rapid and full recovery. SP was re-injected every 5 min. Figure 4A shows the changes in fluorescence ratio of the YC3.6 sensor after injections of 500 pM SP. The inset of Figure 4A shows a linear correlation of the calcium signal maxima to the log of the concentrations between 0.6 and 10.3 ng/µL NK1 plasmid DNA. Spots with the lowest NK1 plasmid DNA concentration of 1% of total DNA yielded a calcium response that was only ~4-fold less than the response of spots with a 50-fold higher NK1 plasmid DNA concentration. In a second array (Figure 4B), we used an NK1 plasmid DNA concentration of 3–30 ng/µL relative to 33 ng/µL total DNA. This array was exposed to three sequential injections of 500 pM SP. While there was response variation (3%–10% in peak height) between spots, at the spot level, responses were highly reproducible and quickly returned to baseline after each stimulation. Yet, at all NK1 plasmid DNA concentrations, except the spots with ~3 ng/µL NK1 plasmid DNA, a slight decrease in peak height was observed for subsequent injections at that high SP concentration. The largest decrease in signal, about 20% of peak height, was found in the spots where the highest NK1 plasmid DNA concentration has been used (Figure 4C). Not surprisingly, two-way ANOVA showed that both NK1 concentration and injection number were highly significant (*p* < 1 × 10^−4^).

Using this array, we made dose response curves by sequentially injecting 3 × 50 pM, 3 × 100 pM and 2 × 500 pM SP onto one array and 3 × 500 pM, 3 × 1 nM and 1 × 5 nM SP onto a second one. The averages of peak heights are shown in Figure 4D. We observed increasing peak heights for spots with increasing NK1 plasmid DNA concentrations up to 500 pM SP. Thereafter, the trend reversed for spots with 15.8 ng/µL or more NK1 plasmid DNA. After the 500 pM SP injection, the subsequent injections of 1 and 5 nM SP resulted in increasingly lower peak heights for those spots with high NK1 plasmid DNA concentrations. In contrast, a single injection of 5 nM SP on a freshly-prepared array resulted in the highest peaks from spots with the highest NK1 plasmid DNA (data not shown). The lowered maxima for the final 5 nM injection in spots with 15.8 ng/µL or more NK1 plasmid DNA were a result of the injection sequence of this experiment.

### 3.5. Specific Receptor Responses to Repeated Challenges

**Figure 5:**
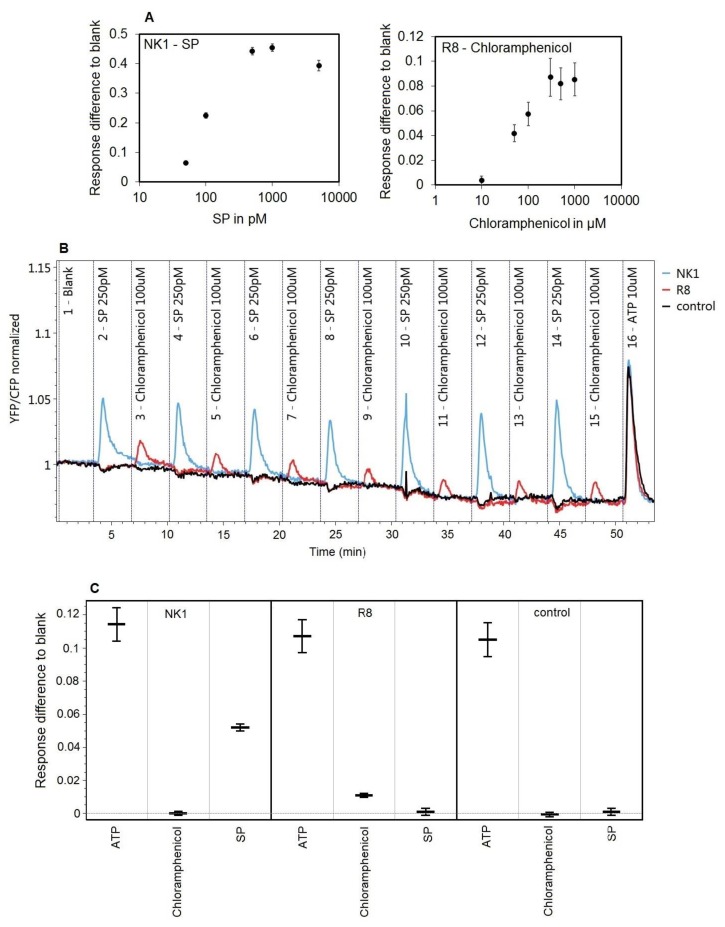
Repeated challenges of two distinct GPCRs by a series of samples. (**A**) Dose-response curves of both NK1 and TAS2R8 for SP and chloramphenicol on a different array. (**B**) Average response profile of NK1, TAS2R8 and the no receptor control of the whole injection series. The first injection is a blank injection of assay buffer; Injections 2–15 are sample exposures of 30 s to either SP at 250 pM or chloramphenicol at 100 µM; and the last Injection 16 is ATP at 10 µM. Response profiles are an average of about 60 spots per spot type. For easier visual interpretation, responses were normalized by dividing the CFP and YFP signals by the average of the first images before sample exposure. (**C**) Estimates and confidence intervals for receptor-specific responses (peak heights) to injections, compared to the blank injection.

In the previous section, we described that within a certain receptor DNA concentration and amount of ligand, repeated injections produce similar receptor responses. Receptor responses are reproducible with repeated injections. In this section, we show how receptors respond to a series of repeated sample injections in a large array containing >60 replicates of three types of transfected spots; one carrying the NK1 receptor, one the bitter R8 receptor and one the control with mock DNA, all printed at 25 ng/µL for high sensitivity and all with 25 ng/µL YC3.6 coding DNA of the calcium sensor. Reverse transfection was performed with HEK293 cells that stably expressed the chimeric Galpha16GUST44 G-protein to allow calcium signaling from bitter receptors [33]. The ready array was mounted into the flow cell, and dose response curves were determined for each receptor for activation by chloramphenicol and SP (Figure 5A). At a selected non-saturating concentration, ligands for both receptors were then intermittently injected every 3 min during a period of >50 min (Figure 5B). We observed specific and reproducible response patterns for each receptor/ligand combination relative to the control. Figure 5C summarizes receptor-specific responses to specific injections, expressed as differences with the response of the blank injection. NK1 gives a significant response to SP, but not to chloramphenicol; R8 shows the reverse behavior. All spots including the control response to the last injection of ATP because it triggers the P2Y receptor, which is natively expressed by the HEK293 host cells [36].

## 4. Discussion

In this study, we have laid out the methodology and critical steps to use reverse transfected cell arrays in microfluidic flow cells [25,26] for the robust, quantitative screening of GPCR libraries.

Our first experiments were aimed at studying reverse transfection and expression efficiency and revealed considerable differences in protein expression levels between individual cells (see Video S2). These differences may partially derive from uneven deposition of DNA on the glass surface or random differences in DNA uptake between cells [37], but most likely mainly reflect the well-known effect of the variation in cell cycle within the cell population used during reverse transfection [13]. Before plasmid DNA is taken up by the nucleus, it can be continuously broken down in the cytoplasm [14], and trafficking of plasmid DNA to the nucleus is only optimal during mitosis. As a result, the sub-population of cells transfected in their late S or G2 phases will transfer higher amounts of plasmid DNA to the nucleus during the M phase than those cells transfected during the G1/G0 phases. Therefore, to generate a more homogeneous and high gene expression, one could either synchronize the cell cycle prior to reverse transfection [38,39], or alternatively, add nuclease inhibitors during the reverse transfection process [40] to reduce plasmid breakdown. In our experiments, we did not synchronize the cells because the transfected DNA was well expressed and allowed sufficient receptor readout in our cell line. However, with other cell lines, smaller spots or poor signals, it may be advisable to synchronize cells for higher and more homogeneous gene expression.

In large reverse-transfected cell arrays, the variation in individual cell expression was captured in the variation of pixel intensities. To analyze these large cell arrays, we introduced thresholds to separate fluorescent pixels from background and over-exposed pixels. Resulting spots were defined as the collection of pixels with values between the upper and lower thresholds. Using this method, we examined the optimal DNA concentration in the print solution by assessing the expression of fluorescent proteins and found a non-linear increase in average fluorescence per spot with increasing total DNA concentration. Examination of the effect of DNA concentration in the print solution on protein expression revealed that, within a certain range, the total DNA concentration has a much greater influence on protein expression than the relative specific DNA concentration. The 10-fold increase in fluorescence signal that we observed could be accounted for almost entirely by the quantity of total DNA [41]. By contrast, when keeping the total DNA concentration constant, we observed a linear increase in fluorescence relative to the specific DNA concentration. A similar linear increase of fluorescent protein expression with constant total DNA was obtained by Woodruff et al. [19]. Overall, the results indicate that, in the tested concentration range, the efficient nuclear uptake of DNA is controlled more by the total DNA concentration than by the specific DNA concentration, thus suggesting strong differences in DNA survival. Furthermore, other studies have shown that adding non-coding plasmid DNA can improve expression efficiency by serving as a protection buffer against nucleases in the cytoplasm [41].

The goal of our experiments was to develop a versatile microfluidic system to screen GPCR libraries by sequentially injected samples. Co-transfection with the fluorescent cytosolic calcium ion sensor Yellow Cameleon YC3.6 enabled the monitoring of transient calcium ion changes and discrimination of transfected and non-transfected cells on the cell array. Additionally, co-transfection allowed us to tune the expression of both genes independently and to analyze the effect of receptor coding DNA concentration separately from the calcium sensor concentration. We observed that the lowest concentration of specific, NK1-encoding DNA, 1% of total DNA (0.3 ng/µL), still yielded sufficient gene expression for a significant increase in intracellular calcium in response to 500 pM SP. This suggests that sufficient numbers of intact plasmids reach the cell nucleus to facilitate NK1 gene expression even at those ratios. Studies have shown that during a transfection event, individual cells can take up about 10^5^–10^6^ copies of a plasmid [16]. However, as discussed earlier, plasmids also need to reach the nucleus in order to be expressed, but are prone to rapid degradation by nucleases naturally present in the cytoplasm [14]. The nuclear plasmid copy number was determined by Cohen et al. in 2009 [15] to range 40-fold from 75–3000 copies per nucleus, and they also demonstrated a direct correlation between nuclear plasmid concentration and gene expression. We used a 50-fold difference in NK1 receptor DNA concentration in the spot mixes and found that the lowest concentration NK1 coding DNA resulted in only a four-fold decrease in the calcium ion signal. It will be interesting in the future to use the reverse-transfected cell array format to evaluate the effect of receptor expression level on the signaling function of other GPCRs or ion-channels, because the highest expression level may not be optimal for gene function. Similarly, these arrays are highly suitable to evaluate permutated cofactor genes and to discover orphan receptor function.

We subjected the NK1 receptor cell array to sequential injections of ligand and monitored the calcium responses. This revealed that spots with higher amounts of NK1 plasmid DNA and higher response maxima were subject to losing signal strength more rapidly compared to spots with lower amounts of NK1 plasmid DNA. Such declining receptor responses are generally known for GPCRs and are thought to be caused by receptor internalization and recycling after (over-)activation [42,43]. Scarce endogenous co-factors of the signaling pathway (G-proteins, beta-arrestins, etc.), specifically associated with the activated receptors, may be depleted more rapidly in the presence of a high receptor density during this process of internalization and possibly explain our observations [28,44]. Therefore, by controlling the GPCR DNA concentration in reverse transfection, the sensitivity and robustness (a reproducible response to repeated challenges) of the receptor response can be optimized. This is shown in the last experiment where we have exposed two different receptors sequentially to alternating samples. These receptors proved to be highly specific for their ligand since no cross-reaction was observed and responses were highly reproducible at the given non-saturating concentrations. The analysis required an approach where inter-spot variation was eliminated, focusing only on comparisons within the time profile of individual spots. The mixed-model approach taken here, where each spot has a random intercept, provides a simple and natural way of fitting these data, demonstrating reproducible responses for sequential injected samples.

Overall, the GCPR library screening platform presented here is complementary to multiwell-based systems as it is simple, fast and efficient for screening receptor libraries with small amounts of ligand (see Video S1). It is perhaps less suitable for screening compound libraries because it depends on sequential rather than parallel injection. Previously, reverse transfection arrays encoding GPCR libraries were printed in static multiwell formats [23,24] and not yet as part of a microfluidic platform. We believe that the microfluidic properties and the potentially very large size of the arrays creates significant potential to make this system a useful high throughput and high content screening tool for GPCR and ion-channel libraries. A single sample exposure of 50–150 microliters for 30 s (depending on flow speed and flowcell volume) can reach hundreds of different receptors in parallel. This screening platform is in that sense complementary to systems with multiple micro-chambers that are independently addressed [19,20]. On-chip corrections for fluorescence quenching and sample fluorescence could be implemented with Cameleon mutants that do not bind calcium for example. Receptor saturation levels could be monitored by including calibration injections. Cameleon expressing control spots lacking recombinant receptors can be used to monitor the build-up of intracellular calcium in time and endogenous cell responses and other artefacts affecting the intracellular calcium ion concentrations.

## 5. Conclusions

In conclusion, this paper has studied the main parameters for optimization of reverse transfection cell arrays for the detection of GPCR signaling in a microfluidic system. We observed an intrinsic, large variation in expression levels between the cells of one array spot. On optimization of protein expression efficiency, we found that the total DNA concentration is a critical parameter to achieve high expression levels. Functional co-transfections were performed showing a fine-tuning of receptor expression levels by changing the input plasmid DNA concentration. Concentrations down to 1% of total DNA still yielded specific receptor responses. Finally, repeated receptor challenges showed a highly reproducible signal. These findings provide a confident outlook on introducing this system as a novel high throughput GPCR library screening tool.

## Supplementary Materials

The following are available online at http://www.mdpi.com/1424-8220/18/2/602/s1: Video S1. Animation of the preparation, measurement and analysis of GPCR arrays. Video S2. Reverse transfection time laps movie. Supplemental Figure S1. Overview of slide preparation and slide measurement. Figure S2. Printed DNA array before and after reverse transfection. Figure S3. Fluorescence image of a large reverse-transfected cell array.
